# Ship Segmentation and Georeferencing from Static Oblique View Images

**DOI:** 10.3390/s22072713

**Published:** 2022-04-01

**Authors:** Borja Carrillo-Perez, Sarah Barnes, Maurice Stephan

**Affiliations:** German Aerospace Center (DLR), Institute for the Protection of Maritime Infrastructures, Fischkai 1, 27572 Bremerhaven, Germany; sarah.barnes@dlr.de (S.B.); maurice.stephan@dlr.de (M.S.)

**Keywords:** ship dataset, instance segmentation, ship georeferencing, homography

## Abstract

Camera systems support the rapid assessment of ship traffic at ports, allowing for a better perspective of the maritime situation. However, optimal ship monitoring requires a level of automation that allows personnel to keep track of relevant variables in the maritime situation in an understandable and visualisable format. It therefore becomes important to have real-time recognition of ships present at the infrastructure, with their class and geographic position presented to the maritime situational awareness operator. This work presents a novel dataset, ShipSG, for the segmentation and georeferencing of ships in maritime monitoring scenes with a static oblique view. Moreover, an exploration of four instance segmentation methods, with a focus on robust (Mask-RCNN, DetectoRS) and real-time performances (YOLACT, Centermask-Lite) and their generalisation to other existing maritime datasets, is shown. Lastly, a method for georeferencing ship masks is proposed. This includes an automatic calculation of the pixel of the segmented ship to be georeferenced and the use of a homography to transform this pixel to geographic coordinates. DetectoRS provided the highest ship segmentation mAP of 0.747. The fastest segmentation method was Centermask-Lite, with 40.96 FPS. The accuracy of our georeferencing method was (22 ± 10) m for ships detected within a 400 m range, and (53 ± 24) m for ships over 400 m away from the camera.

## 1. Introduction

### 1.1. Maritime Situational Awareness and Ship Monitoring

Research in the field of maritime safety and security concentrates on the development, testing and validation of innovative systems for the assessment of the status of maritime infrastructures. One aspect of this is in the development of maritime situational awareness systems to quantitatively determine the protection status of infrastructures in real-time and execute measures to respond to threats (e.g., major accidents, natural catastrophes, terror attacks, organised crime) [1]. An automatic and meaningful awareness of the maritime situation requires instruments and sensors that are able to recognise elements of interest to propose suitable measures to the user and authorities [2].

Real-time ship monitoring at ports is one of today’s most challenging tasks for Vessel Traffic Services (VTS) [3], and great efforts are being made using Automatic Identification Systems (AIS) [4]. The International Maritime Organisation (https://www.imo.org/en/OurWork/Safety/Pages/AIS.aspx, accessed on 16 February 2022) requires that every ship and vessel with 300 or more gross tonnage carries AIS transceivers, which must transmit information such as a unique identification number, ship type, position, course and speed, in the form of encoded radio messages. The AIS tracking system allows VTS and the surrounding ships to be aware of the marine traffic in the area and to perform tasks such as collision avoidance or search and rescue. However, the AIS messages are only transmitted by ships in intervals from 2 to 10 s while underway, and up to 6 min in static position (https://www.navcen.uscg.gov/?pageName=AISMessages, accessed on 16 February 2022). This leaves room for real-time systems to analyse the situation several times per second and therefore help to prevent or respond to complications. Moreover, since it is an open standard, AIS presents some vulnerabilities to cyber threats, which include spoofing, highjacking attacks or denial of service [5,6,7,8,9].

Optical camera systems thus become a good choice for the rapid assesment of ship traffic. Due to their availability, affordable price and uncomplicated deployment, they can serve as support in tasks of ship monitoring. When placed with elevation and an oblique view of the water, they allow an excellent perspective of the situation. Personnel might, however, not be able to effectively keep track of everything relevant due to the amount of video screens and information that need to be assessed [10]. It therefore becomes important to have automatic recognition of ships present at the infrastructure, with their class and position presented to the maritime situational awareness operator in a more understandable format, for example on a map.

### 1.2. Ship Segmentation

Deep-learning-based object recognition is one of most well studied computer vision topics and can be applied to recognise ships in images from optical cameras.

One of the trends in the field is to obtain the mask of objects detected with segmentation, which is referred to by the name of instance segmentation. This technique allows the extraction of further information relating to the recognised objects, such as their position on Earth’s coordinate system, referred to as georeferencing. Georeferencing can be better inferred from the segmented mask of an object than from a sourrounding bounding box, which usually contains a lot of unnecessary background.

Instance segmentation is a type of supervised deep learning problem, and datasets for training are needed. There are some general-purpose benchmark datasets, such as COCO [11]. The images available in the general-purpose datasets do not suit specific tasks of ship recognition with the precision required by a maritime awareness application. Real-world maritime situations require varied ship data with precise annotations, that should include the ship class, mask and further features, such as geographic coordinates. Amongst works in the literature that deal with ship detection with optical monitoring views, available datasets are the Singapore Maritime Dataset (SMD) [12], Seaships7000 [13] and the dataset presented by Chen et al. [14]. However, these datasets lack the required annotations to perform instance segmentation, contain low ship variety, and do not have a favourable oblique view, which means that georeferencing becomes difficult to perform. We have not found a public dataset that contains oblique view images of a maritime infrastructure and mask annotations of several types of ships for the exploration, evaluation and development of instance segmentation methods.

State-of-the-art instance segmentation methods with robust results on the COCO dataset are Mask-RCNN [15] and DetectoRS [16]. State-of-the-art methods for real-time applications have been developed in YOLACT [17] and Centermask [18]. Existing works evaluate object detection methods in maritime environments on their private ship detection datasets [19,20]. Nita et al., in [21], tackle the task of ship instance segmentation without a real-time approach, using only Mask-RCNN [15] on their private dataset. A comparison of state-of-the-art ship instance segmentation methods from maritime oblique view images, with a focus on robust and real-time methods, and a public dataset, has not been found.

### 1.3. Ship Georeferencing

Once ships have been detected and segmented, georeferencing is required to provide their real-time location to a situational awareness system.

General-purpose object georeferencing has been studied primarily for airborne applications [22,23,24] and autonomous driving [25]. In the field of ship georeferencing, existing works made use of radar [26], remote sensing using synthetic aperture radar [27] and AIS [28]. Helgesen et al., in [29], proposed a pipeline for ship detection and georeferencing using their private dataset with oblique view images of the water. They use the pinhole camera calibration transformation matrix proposed in [30] to georeference bounding boxes of detected ships. For this, their approach requires previous knowledge of the camera, such as location, elevation, field of view and tilt angle of the lense. Moreover, their study is limited to a maximum range of 400 m between the camera and the ship. An extensive analysis of a methodology for ship georeferencing using oblique view images where previous knowledge of the camera is not required has not been found.

### 1.4. Proposed Work

The aim of this work is to address the gaps in the field of ship segmentation and georeferencing from oblique view images to advance the development of maritime situational awareness systems with higher levels of automated information extraction. Our proposed contributions can be summed up in the following three statements.

First, we present the creation of a novel dataset, ShipSG, for ship segmentation and georeferencing using static oblique view images. This dataset contains mask annotations of the ships present in the images along with their corresponding class, positions (latitude, longitude), and lengths. The dataset was created using two cameras at a port location, and the geographic ship positions were obtained using AIS data. To the best of our knowledge, this is the first dataset of its kind and will be publicly available (https://dlr.de/mi/shipsg, accessed on 16 February 2022).

Second, we explore four instance segmentation methods to recognise ships with the ShipSG dataset. Two as a baseline for robust instance segmentation, Mask-RCNN [15] and DetectoRS [16], and two capable of real-time processing, YOLACT [17] and Centermask-Lite [18]. The goal is to find which of the four provides the best precision for ship segmentation, and which method provides the best trade-off between precision and inference speed. We also provide an approximation of how well these methods generalise after training with the ShipSG dataset on the aforementioned datasets SMD [12], Seaships7000 [13] and the dataset by Chen et al. [14].

Third, we propose a methodology for the automatic georeferencing of ship masks. We automatically calculate the pixel to be geoferenced from the segmented masks provided by the previous instance segmentation step. The georeferencing method we propose is based on the use of a homography matrix to transform pixels from the ShipSG images to geographic latitudes and longitudes.

The following sections of this paper are organised as follows. Section 2 presents the creation of the ShipSG dataset and its content. Also discussed are the selected instance segmentation methods and our proposed ship georeferencing method. Section 3 shows the results of each instance segmentation method along with an analysis of our ship georeferencing method. We present the results in Section 4, followed by our conclusion in Section 5.

## 2. Materials and Methods

### 2.1. The ShipSG Dataset

**Figure 1:**
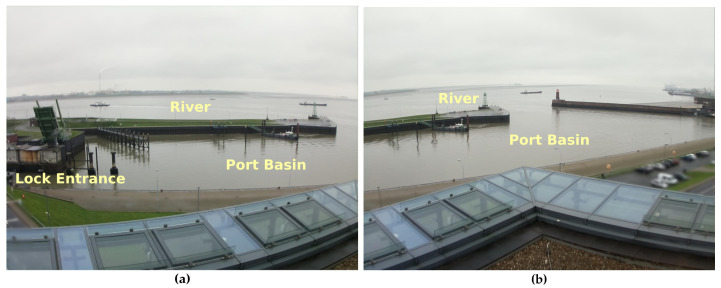
View of each camera used for ShipSG data collection, showing the river, port basin and lock entrance. (**a**) View of first camera. (**b**) View of second camera.

The ShipSG dataset (https://dlr.de/mi/shipsg, accessed on 16 February 2022) was collected using two cameras located at the Fischereihafen-Doppelschleuse lock, part of the port of Bremerhaven, Germany. The cameras have partly overlapping fields of view, facing the port basin, in order to observe the maritime activity at the entrance of the lock and at the river where the port is located (see Figure 1). The port basin is within a 400 m range of the cameras. The range of the river is over 400 m, where ships can be seen up to approximately 1200 m away from the cameras. The acquisition of images took place in Autumn 2020 during daylight hours with sunny, cloudy, windy and rainy weather conditions. The tidal range was between 3 and 4 m (https://gezeitenfisch.com/de/bremen/bremerhaven-doppelschleuse, accessed on 16 February 2022). Vehicles and people appearing within the images were anonymised since they are not of interest for this work.

To obtain information relating to ships present within each image, we accessed AIS position and static messages (https://www.navcen.uscg.gov/?pageName=AISMessages, accessed on 16 February 2022) from AISHub (https://www.aishub.net/, accessed on 16 February 2022). The former is sent by ships in intervals between 2 and 10 s, and the latter in intervals of 3 and 6 min [4]. These messages contain, amongst other fields, the ship position (latitude and longitude in decimal degrees) and the ship length (in meters). We used these two fields to annotate the ships in the images of the dataset. The AIS ship position is used as a ground truth for our georeferencing method and the ship length is used to study how our georeferencing method changes with the ship length. In order to label data, we accessed the timestamp of each image and searched for the AIS message which has the most similar timestamp and a position which lies within the field of view of the cameras. We defined 100 milliseconds as the maximum offset between the image and AIS timestamp so that the position of the ship seen in the image corresponds as close as possible with the position contained in the AIS message. Since a short offset is used, and due to the fact that ships send AIS messages with a time period of seconds, this leads to only one AIS reference of a ship per image. We discarded images in which the timestamp could not be related to any AIS message timestamp. A total of 3505 images were found with an AIS message corresponding to one of the ships within the image.

**Figure 2:**
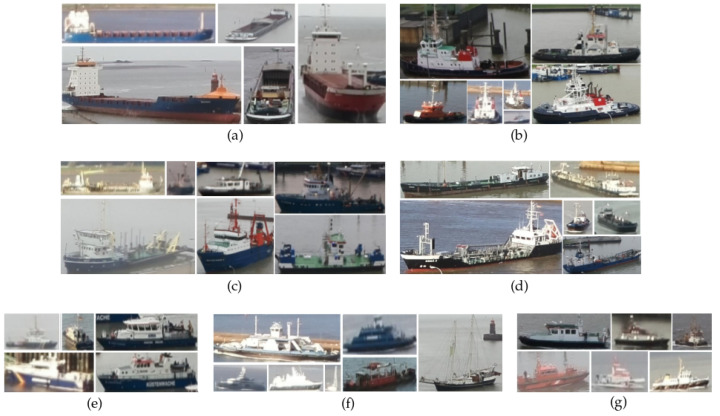
Examples extracted from the dataset that show the seven ship classes. Each class contains a variety of sizes and orientations of the ships. (**a**) Cargo , (**b**) Tug, (**c**) Special 1, (**d**) Tanker, (**e**) Law Enforcement, (**f**) Passenger/Pleasure, (**g**) Special 2.

We designated the ship classes for the dataset based on an observation of their purpose and visual similarities. Examples of each ship class are shown in Figure 2 and are described as follows:

- Cargo: All types of cargo ships.
- Law Enforcement: Police watercrafts and coast guard ships.
- Passenger/Pleasure: Ferries, yachts, pleasure and sailing crafts.
- Special 1: Crane vessels, dredgers and fishing boats.
- Special 2: Research and survey ships, search and rescue ships and pilot vessels.
- Tanker: All types of tankers.
- Tug: All types of tugboats.

**Figure 3:**
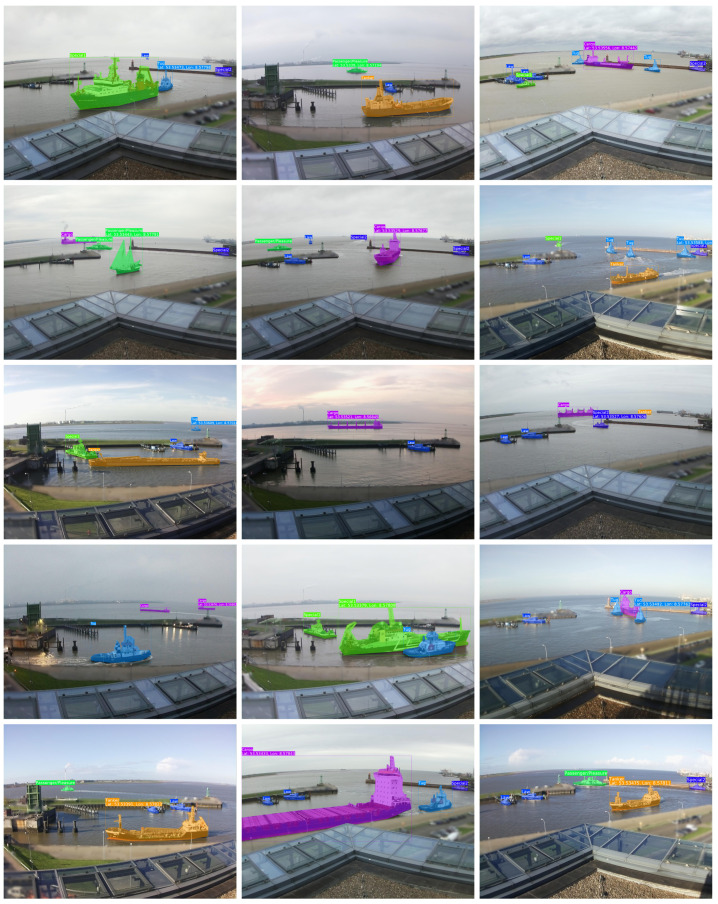
Visualisation of ShipSG dataset samples with annotated ship masks and classes, and one ship position per image.

For the task of instance segmentation, annotations of ship masks are needed as input for algorithm training. We manually annotated the ship masks within each image with their corresponding class using the LabelMe software [31]. Figure 3 illustrates samples of our dataset with the annotated masks.

We used the definition of small, medium and large mask area scales that were introduced for the COCO dataset [11], and have the following values:

$$Mask\;Area\;Scale=\left\{\begin{array}{cc} \mathrm{Small}, & \mathrm{if}\;area\leq 32^{2}\;\mathrm{pixels},\\ \mathrm{Medium}, & \mathrm{if}\;32^{2}<area\leq 96^{2}\;\mathrm{pixels},\\ \mathrm{Large}, & \mathrm{if}\;area>96^{2}\;\mathrm{pixels} \end{array}\right.$$

These area scales are later used in this work to measure the performance of instance segmentation algorithms. Table 1 lists the number of masks annotated in the dataset per class, as well as the number of masks per area scale in pixels. In total, 11625 masks were annotated.

In summary, the ShipSG dataset contains:

- 3505 images from the two cameras.
- 11,625 annotated ship masks grouped in seven classes with COCO format [11]. The AIS ship type will also be shared so that future users can create their own classes.
- 3505 geographic positions, consisting of the latitude and longitude of one of the masks within each image.
- 3505 ship lengths, one per geographic position annotated.

The authors of this paper intend to provide the dataset to the public (https://dlr.de/mi/shipsg, accessed on 16 February 2022). To the best of our knowledge, this dataset is the first of its kind dedicated to ship segmentation and georeferencing, and which is available to the public. It was not possible to share the MMSIs associated with the AIS messages for the ShipSG dataset due to the underlying principles of the privacy policy implemented when performing data acquisition. This policy was composed by the German Aerospace Center (DLR) and implements the General Data Protection Regulation (EU) 2016/679 [32].

### 2.2. Instance Segmentation Methods Selected

In order to explore the task of instance segmentation using our dataset, four state-of-the-art methods were selected. Two as a baseline for robust instance segmentation and two capable of real-time processing, which are described in Section 2.2.1 and Section 2.2.2, respectively. The comparison of these methods follows the standard metrics used by the COCO dataset [11], based on the well-known average precision (AP). The mean average precision (mAP) is the calculated mean of all the AP of the classes present. As a metric of speed during inference, frames per second (FPS) are considered.

#### 2.2.1. Robust Instance Segmentation Methods

Mask R-CNN [15] is a two-stage algorithm that was developed as an extention of the object detector Faster R-CNN [33]. In the first stage, with the region proposal network [33], multiple object candidates are proposed. In the second stage, the region of interest pooling extracts features from each candidate and performs the classification of the object and the regression of the bounding box. In Mask R-CNN, a fully convolutional network was added to regress the mask from the detected bounding boxes. This method is one of the most popular in the field of instance segmentation for its robustness. With the ResNeXt-101 backbone [34], it achieves a mask mAP of 0.375 on the COCO dataset.

DetectoRS [16] is a multi-stage network that proposed a recursive feature pyramid [35] to include additional feedback connections from feature pyramid networks into the bottom-up backbone layers. Its authors also propose the convolution of features by looking twice at the input with different atrous rates and then to combine the outputs, which is referred to as switchable atrous convolution. This method is a state-of-the-art instance segmentation method, and with ResNet-50 as the backbone [36], it achieves a mask mAP of 0.444 on the COCO dataset.

#### 2.2.2. Real-Time Instance Segmentation Methods

YOLACT [17] emerged as one of the first real-time and one-stage instance segmentation methods. It uses an independent fully convolutional network [37] to produce prototype masks and a parallel branch to calculate mask coefficients for each predicted anchor box, which are filtered using non-maximum supression. Both branches are combined by cropping and thresholding the prototyped masks with the filtered anchor box. On the COCO dataset, with ResNet-101 as the backbone [36] and 700 × 700 pixels as an input size, it achieves an mAP of 0.312 and an inference speed of 23.4 FPS.

Centermask [18] is a one-stage method. It makes use of the fully convolutional one-stage object detector [38], and introduces a spatial attention-guided mask branch, which is paired with the object detector to suppress the pixels that do not belong to the mask on the regions proposed as boxes. Specifically, for our work, we selected Centermask-Lite, a downsized version of the original which is better suited for real-time applications. The authors of Centermask introduced in [39] a novel backbone, VoVNet, where instead of adding residual shortcuts every second feature map, as is done in ResNet, features are concatenated only once in the last feature map. With VoVNet-39 as backbone, Centermask-Lite achieves a mask mAP of 0.363 and 35.7 FPS on the COCO dataset.

### 2.3. Ship Georeferencing Using Homography

Once the ships are segmented using an instance segmentation method, georeferencing is required to provide their location to the situational awareness system.

The use of homography, an isomorphism between projective spaces, is well established in the computer vision field to transform points from the same planar surface captured by two perspectives, which up to now has not been deeply studied in the context of ship georeferencing.

We tested the use of a homography qualitatively for ship georeferencing for maritime anomaly detection environments in [40]. The present work will expand upon this and make an in depth quantitative study of the use of homographies for ship georeferencing.

We take advantage of the static view of the cameras and perform a transformation by calculating the homography between the camera pixel coordinates $\left(C_{x},C_{y}\right)$ and Earth’s geographic latitude and longitude (φ,λ) in decimal degrees. Since the ground surface area captured by the cameras is small enough with respect to the Earth’s local curvature, it is approximated by its tangent plane.

A homography, expressed as the matrix *H*, used to perform the transformation is shown in Equation (1), where the unknown parameters $h11,h12,\ldots ,h_{32}$ are calculated using *n* number of pixel pairs $\left(C_{x},C_{y}\right)$ and geographic latitude and longitude pairs (φ,λ) as shown in Equation (2). Using *n* = 4 pairs of pixel coordinates and their corresponding geographic positions would suffice to solve the eight unkown parameters of Equation (2) ($h11,h12,\ldots ,h_{32}$). Having more pairs would result in more than one solution for each parameter of the equation. Therefore, *n* > 4 is preferred, allowing the optimal solution for each unknown parameter to be calculated using least squares.

(1) $$\begin{array}{c} \left[\begin{array}{c} \varphi \\ \lambda \\ 1 \end{array}\right]=H\left[\begin{array}{c} C_{x}\\ C_{y}\\ 1 \end{array}\right]=\left[\begin{array}{ccc} h_{11} & h_{12} & h_{13}\\ h_{21} & h_{22} & h_{23}\\ h_{31} & h_{32} & 1 \end{array}\right]\left[\begin{array}{c} C_{x}\\ C_{y}\\ 1 \end{array}\right]. \end{array}$$

(2) $$\begin{array}{c} \left[\begin{array}{cccccccc} C_{x_{1}} & C_{y_{1}} & 1 & 0 & 0 & 0 & -\varphi_{1}\cdot C_{x_{1}} & -\varphi_{1}\cdot C_{y_{1}}\\ 0 & 0 & 0 & C_{x_{1}} & C_{y_{1}} & 1 & -\lambda_{1}\cdot C_{x_{1}} & -\lambda_{1}\cdot C_{y_{1}}\\ C_{x_{2}} & C_{y_{2}} & 1 & 0 & 0 & 0 & -\varphi_{2}\cdot C_{x_{2}} & -\varphi_{2}\cdot C_{y_{2}}\\ 0 & 0 & 0 & C_{x_{2}} & C_{y_{2}} & 1 & -\lambda_{2}\cdot C_{x_{2}} & -\lambda_{2}\cdot C_{y_{2}}\\ & & & & & \vdots \\ C_{x_{n}} & C_{y_{n}} & 1 & 0 & 0 & 0 & -\varphi_{n}\cdot C_{x_{n}} & -\varphi_{n}\cdot C_{y_{n}}\\ 0 & 0 & 0 & C_{x_{n}} & C_{y_{n}} & 1 & -\lambda_{n}\cdot C_{x_{n}} & -\lambda_{n}\cdot C_{y_{n}} \end{array}\right]\left[\begin{array}{c} h_{11}\\ h_{12}\\ h_{13}\\ h_{21}\\ h_{22}\\ h_{23}\\ h_{31}\\ h_{32} \end{array}\right]=\left[\begin{array}{c} \varphi_{1}\\ \lambda_{1}\\ \varphi_{2}\\ \lambda_{2}\\ \vdots \\ \varphi_{n}\\ \lambda_{n} \end{array}\right] \end{array}$$

The *n* pairs have to be selected manually in advance to create *H*. Multiple pairs distributed throughout the geographic and image area of interest should be selected, for a better adjusted *H*. For $\left(\varphi_{1\ldots n},\lambda_{1\ldots n}\right)$ we used the geographic annotation that each image contains, as explained in Section 2.1, and for $\left(C_{x_{1\ldots n}},C_{y_{1\ldots n}}\right)$ the pixel coordinates of the corresponding ship in the corresponding image. To manually select this pixel, we observed the placement of the navigation antenna on each ship, since this is the element which provides the geographic location in the AIS messages. This antenna is usually located on the bridge or wheelhouse of the ship. We therefore selected the pixel that intersects the ship hull at the antenna location and the water underneath as the pixel corresponding to the latitude and longitude gathered with AIS.

**Figure 4:**
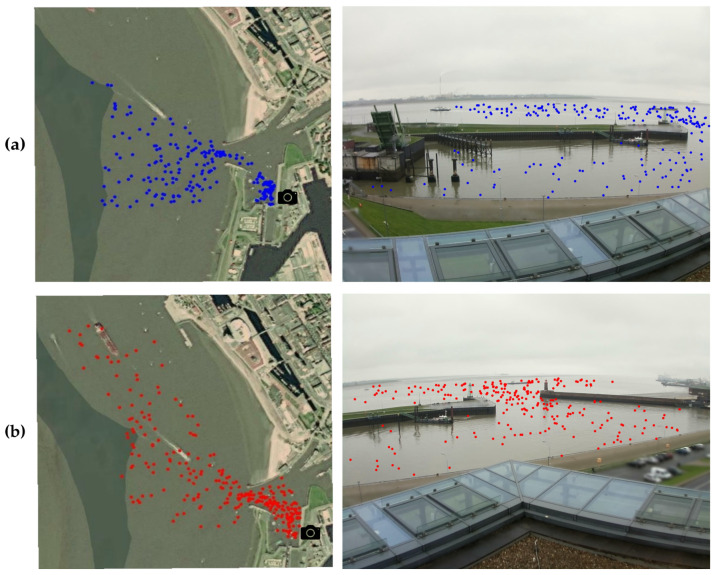
Reference pairs to create the homographies. The coloured dots to the left show the latitudes and longitudes obtained from the AIS messages on a map. To the right, the counterpart pixel coordinates are displayed, annotated by hand. The black icons show the location of the cameras. (**a**) First camera pairs for homography creation, with blue dots. (**b**) Second camera pairs for homography creation, with red dots.

We took *n* = 200 samples of the training set images to create the homographies of both cameras (see Figure 4), and solved Equation (2) to obtain *H*. The validation set is later used to quantitatively analyse how well this method performs.The separation of homographies by high or low tides was not found to provide a significant improvement in results.

**Figure 5:**
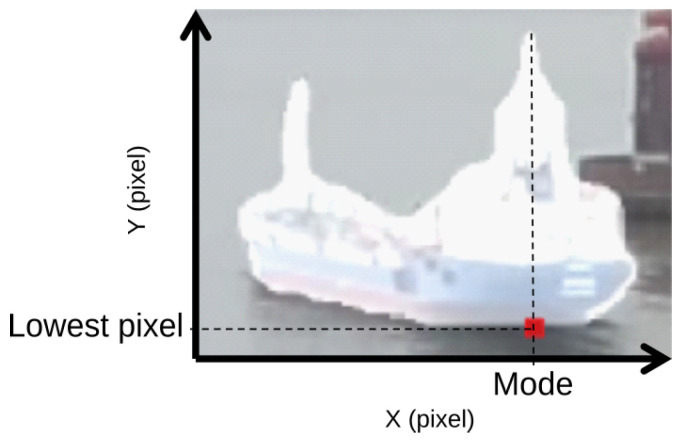
Example of segmented ship mask with calculated pixel to be georeferenced (in red, enlarged for visualisation).

Once the homographies are created, we then propose a method to automatically calculate the pixel ($C_{x},C_{y}$) of the mask which best represents the ship’s geographic position. This pixel is afterwards georeferenced using Equation (1). We automatically find the pixel which lies at the intersection point between the ship hull and the water below the bridge or wheelhouse, where the navigation antenna of the ship is located. We calculate this pixel to be the lowest of the mask in the vertical direction (*Y*) corresponding to the statistical mode of the horizontal axis (*X*). An example of this procedure can be seen in Figure 5.

## 3. Results

### 3.1. Experimental Evaluation of Instance Segmentation Methods on the Dataset

We split the dataset into two sets of images—training and validation. The training set contains 80% of the dataset, with 2804 images, and the remaining 20% is used for validation, with 701 images. Both sets have a comparable class distribution, as the one shown in the last column of Table 1.

The training and evaluation of the robust methods discussed in Section 2.2.1, Mask R-CNN and DetectoRS, was done using the MMdetection framework from OpenMMLab [41]. The input image size in both cases was 1333 × 800 pixels, and the backbones selected were ResNeXt-101 and ResNet-50, respectively.

For the experimental setup of the real-time methods discussed in Section 2.2.2, our interest was to look for the optimal trade-off between inference speed and AP. Therefore we selected two configurations for each, one with a deeper backbone (more layers) and one with a shallower backbone. The implementation of YOLACT used was the source provided by its authors [17]. The first configuration, YOLACT_550_, uses a smaller input size (550 × 550 pixels) and the lighter ResNet-50 [36] as backbone and which will provide faster inference speed. The second configuration, YOLACT_700_, uses a higher input size of 700 × 700 pixels and a deeper backbone, the ResNet-101 [36], which will provide higher AP. As for Centermask-Lite, we used the source implementation provided by its authors [18], which is implemented on top of the framework Detectron2 [42]. For a faster inference speed, the first configuration, Centermask-Lite_v19_, uses a lighter backbone, VoVNet-19 [39]. The second, Centermask-Lite_v39_, uses the deeper VoVNet-39 [39], for higher AP. Both Centermask-Lite configurations use an input size of 800 × 600 pixels, as per definition by their authors [18].

Table 2 shows a summary of the training configurations. All the methods were initialised with weights pre-trained on the COCO dataset [11]. The Pytorch version used for the four methods was 1.8.1 and the GPU used to train and compute the inference speed was a Nvidia Quadro GV100.

Table 3 illustrates the results per method evaluated. For the evaluation and comparison of these methods, we chose the standard metrics used by the COCO dataset [11]. These are the overall mAP, the mAP at intersection over union 50% (mAP_50_), the mAP at intersection over union 75% (mAP_75_) and the mAP at different mask area scale, i.e., mAP_S_ for small objects, mAP_M_ for medium objects and mAP_L_ for large objects (https://cocodataset.org/#detection-eval, accessed on 16 February 2022). As well as the mAP, we include the class-agnostic mask AP (AP_ca_) which shows that there is not significant class imbalance. As a metric of speed during inference, frames per second (FPS) are considered. The complete results showing each AP per class and per instance segmentation method are shown in Appendix A.

Out of the robust methods evaluated, Mask R-CNN [15] and DetectoRS [16] reach the best mask mAP in all cases compared to the real-time methods. DetectoRS achieves the highest AP_ca_ and mAP, with values of 0.780 and 0.747, respectively. The inference speed of these methods provides 8.50 and 6.62 FPS, respectively.

Comparing the performances of the real-time methods, YOLACT [17] and Centermask-Lite [18], we observe that Centermask-Lite_V39_ achieves the highest AP_ca_ and mAP. Moreover, Centermask-Lite in both configurations performs better with small and medium objects. YOLACT, however, achieves a higher mAP with large objects, 0.751, compared with Centermask-Lite_V39_, 0.661. In terms of inference speed, Centermask-Lite_V19_ achieves the highest FPS value of 40.98.

### 3.2. Generalisation of the Evaluated Instance Segmentation Methods on Other Datasets

It is advantageous for a computer vision architecture to be able to perform well in diverse scenarios, since this indicates that overfitting due to the dataset used has not ocurred.

**Figure 6:**
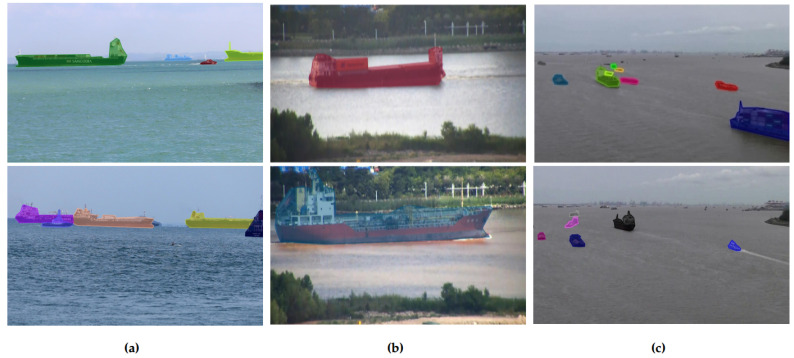
Annotated masks on existing datasets for the study of the generalisation of our models. (**a**) Annotated examples of the SMD [12]. (**b**) Annotated examples of Seaships7000 [13]. (**c**) Annotated examples of the dataset by Chen et al. [14].

To study the ability of the instance segmentation models trained in Section 3.1 to generalise in different scenarios, we analysed their performance with test images from other existing datasets with similar characteristics: SMD [12], Seaships7000 [13] and the dataset by Chen et al. [14]. Since these datasets do not provide ship mask annotations, we annotated the ships present in 100 images of the three datasets and combined them into a mini-dataset with a single class. Samples of this mini-dataset for generalisation can be seen in Figure 6. The annotated content is as follows:

- SMD [12]: Two images per on-shore scene, totalling 80 images.
- Seaships7000 [13]: 12 random images of the dataset.
- Dataset by Chen et al. [14]: Two images per scene, totalling eight images.

Table 4 shows the results after inference on the generalisation mini-dataset. The best AP is achieved by DetectoRS with 0.486. When looking at the AP_50_, we observe that DetectoRS provides a satisfactory value of 0.830. Of the real-time methods, Centermask-Lite_V39_ achieves the best AP, with 0.387, and an AP_50_ of 0.710.These results show that the models trained with ShipSG can predict ships from other datasets with reasonable accuracy.

### 3.3. Experimental Evaluation of Ship Georeferencing

The data collection process, explained in Section 2.3, shows that every image of the dataset contains one ship position taken from AIS messages (latitude and longitude) along with the corresponding pixel annotation. We created two homographies, one per camera, as shown in Section 2.3. We then quantitatively analysed how well the method performs for the task of ship goereferencing.

We took the resulting ship masks from DetectoRS [16] using the 701 images of the validation set, which contains ship images from both cameras. We georeferenced the masks using Equation (1), after following the proposed method described in Section 2.3.

**Figure 7:**
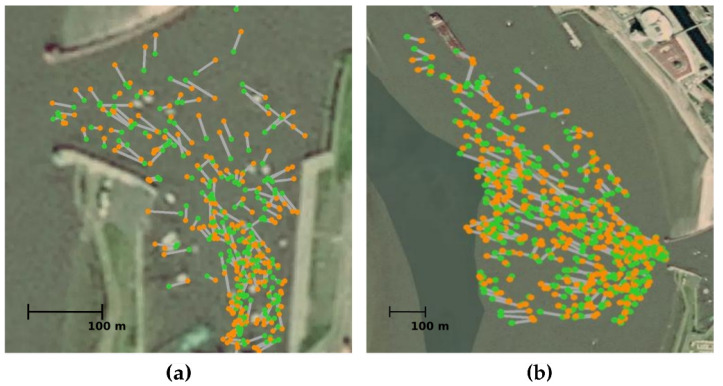
Qualitative ship georeferencing results. (**a**) Port basin. (**b**) River. Green dots show the positions given by AIS. Orange dots show the georeferenced positions using our method). Gray lines join the actual and georeferenced positions.

Figure 7 shows the qualitative results of our proposed ship georeferencing method. As introduced in Section 2.1, we split the field of view into the port basin area and the river area in order to observe the results from both camera ranges to the ships. These are smaller and greater than 400 m, respectively.

We quantitatively compared the true latitudes and longitudes collected with AIS ($\varphi_{AIS}$, $\lambda_{AIS}$) and the homography-georeferenced latitudes and longitudes ($\varphi_{H}$, $\lambda_{H}$). For this comparison, we convert both latitudes and longitudes from decimal degrees to Universal Transverse Mercator (UTM) to express every result in meters. The metrics used to determine how well the technique performs are the following:

- Latitude absolute error (Δφ):
  (3) $$\Delta \varphi =|\varphi_{AIS}-\varphi_{H}|$$
- Longitude absolute error (Δλ):
  (4) $$\Delta \lambda =|\lambda_{AIS}-\lambda_{H}|$$
- Georeferencing distance error (GDE), to measure the distance between true and georeferenced positions. The haversine equation is used instead of euclidean distance, to take into account the radius (R) and therefore curvature of the Earth:
  (5) $$GDE=2\;\cdot R\cdot \arcsin\sqrt{\sin^{2}\frac{|\varphi_{AIS}-\varphi_{H}|}{2}+\cos\varphi_{AIS}\cdot \cos\varphi_{H}\cdot \sin^{2}\frac{|\lambda_{AIS}-\lambda_{H}|}{2}}$$
- Distance root-mean-square error (*DRMSE*) [43], to measure the quadratic mean of all latitude and longitude errors. Due to the squared differences, larger errors are more penalised than small errors:
  (6) $$RMSE_{\varphi }=\sqrt{\frac{1}{k}\Sigma_{i=1}^{k}{\left(\varphi_{AIS_{i}}-\varphi_{H_{i}}\right)}^{2}}$$
  (7) $$RMSE_{\lambda }=\sqrt{\frac{1}{k}\Sigma_{i=1}^{k}{\left(\lambda_{AIS_{i}}-\lambda_{H_{i}}\right)}^{2}}$$
  (8) $$DRMSE=\sqrt{{RMSE_{\varphi }}^{2}+{RMSE_{\lambda }}^{2}}$$

Table 5 shows the quantitative results. For all metrics, a lower value indicates a better result. As expected, our method is more accurate the closer the ships are to the cameras, which is represented by the smaller values of all metrics within the port basin. This can be compared to the river area, where every pixel covers more geographical area, and therefore the error becomes more significant. We consider the mean GDE as the most representative metric, because it directly measures the distance in meters between the actual and estimated positions. This metric reaches (22±10) m inside the port basin and (53±24) m on the river. The DRMSE calculated inside the port basin is 27 m, and 61 m on the river, providing a comparable result to the GDE metric. This indicates that errors larger than the mean GDEs are not common and do not have great impact, showing therefore that the method works consistently.

**Figure 8:**
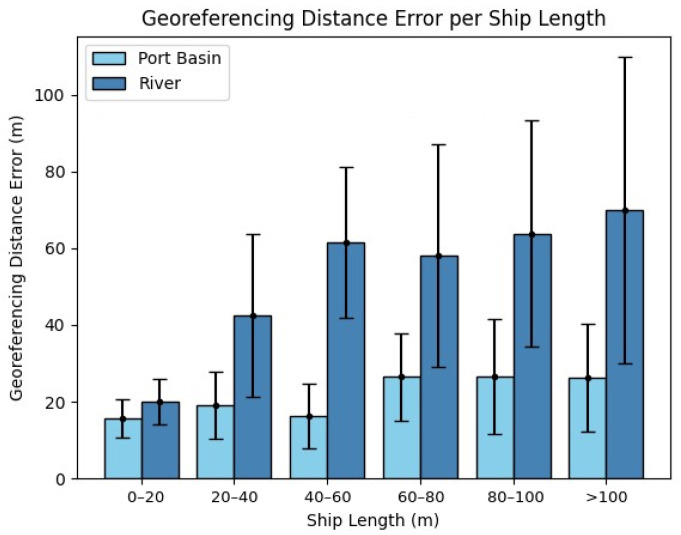
Georeferencing distance error per ship length. GDEs and their uncertainties fall within the bounds of the ship length.

In Section 2.1 it was described that we collected ship lengths along with the ship positions from AIS messages. This was done to observe how the GDE changes with the ship length, as shown in Figure 8. For the smallest ship lengths (0 to 20 m), the GDE within the port basin and river are similar. This shows that, independently from the range between ship and camera, the smaller the ship, the more accurately the method finds the pixel of the mask to be georeferenced.

Range is the largest contributor to the GDE. For ships over 20 m long, there is a significant increase in GDE when viewed at ranges greater than 400 m (at the river). For ranges closer than 400 m (within the port basin), the ship length has less impact on the resulting GDEs than on the river.

Even though the larger and more distant the ship, the more difficult it becomes to find the exact pixel of the mask to be georeferenced, all GDEs are consistent within uncertainties per ship length. This shows that the method provides a reliable estimation, including for larger ships on the river side where every pixel covers more geographical area.

## 4. Discussion

In this work, ShipSG, a novel dataset for ship segmentation and georeferencing, has been presented. This dataset contains 3505 images from a static oblique view, using two cameras with partly overlapping views. A total of 11,625 ship masks were annotated and grouped in seven ship classes. Through the use of AIS messages, acquired simultaneously with the images, we annotated the geographic position (latitude and longitude) and length of one ship per image. To the best of our knowledge, this is the first dataset of its kind. This dataset will be shared with the public (https://dlr.de/mi/shipsg, accessed on 16 February 2022). We split the ShipSG dataset into training (80%) and validation (20%) sets, and explored four state-of-the-art instance segmentation methods for the automatic recognition of the annotated ships. Two robust methods, Mask-RCNN [15] and DetectoRS [16], and two real-time methods, YOLACT [17] and Centermask-Lite [18], were explored. Each real-time method was studied with two configurations, with a lighter and a deeper backbone. After training all methods with the ShipSG dataset, DetectoRS provides the best mAP, of 0.747. The fastest method explored was Centermask-Lite_V19_, with 40.96 FPS. Centermask-Lite_V39_, with the deeper backbone Vovnet-39 [39], provided the best trade-off between mAP and FPS, with 0.644 and 35.25, respectively, which makes it the most suitable candidate for tasks of real-time ship segmentation for a future situational awareness system. YOLACT_700_, however, with ResNet-101 as the backbone [36], performs better with large objects (mAP_L_ of 0.751) when compared with Centermask-Lite (mAP_L_ of 0.661). Since the key aim of this work is to recognise ships at all ranges and with all sizes and classes, future work will focus on improving large object segmentation for Centermask-Lite.

As all instance segmentation methods provided a good mAP, higher than 0.5 in all cases, we tested how well they generalise when segmenting ships of other maritime datasets after training with ShipSG. We annotated the ship masks of a mini-dataset of 100 images from SMD [12], Seaships7000 [13] and the dataset by Chen et al. [14], which are datasets that either did not contain the necessary annotations or are not suitable due to the lack of variety of ships and scenes, but still contain some ships that could be used for testing. DetectoRS still showed the best AP, with 0.486, and AP_50_ of 0.830. Centermask-Lite_V39_ provided the best AP of the real-time methods explored, with 0.387 and AP_50_ of 0.710. It has been shown, therefore, that the methods trained with the ShipSG dataset could be used for inference on other similar maritime scenes.

A method for the automatic geoferencing of ship masks has been presented. The method is based on the use of a homography matrix to transform pixels from the ShipSG images, taking advantage of the static view, to geographic latitudes and longitudes. The homographies, one per camera, were created using the AIS positions of the ships present in the training set images of ShipSG. The georeferenced pixel is chosen to be that which intersects the ship hull and the water below, at the point where the navigation antenna is located on the ship. We also present a method to automatically calculate this pixel.

We quantitatively analysed our proposed method for ship georeferencing at a range closer than 400 m (within the port basin) and farther than 400 m (on the river). As expected, the accuracy of the method is best when the ship is closest to the camera. Furthermore, independently from the range between the ship and camera, the smaller the ship, the more accurately the method finds the pixel of the mask to be georeferenced. The results prove that this is a reliable approach, since the georeferenced pixel is shown to fall within the bounds of the ship (Figure 8) and within the uncertainty of the method (Table 5) when considering both shorter and longer camera ranges.

A future maritime situational awareness tool for ship georeferencing used by, for instance, authorities, would need a series of further improvements to avoid the estimation of the presence of recognised ships in a location where an error could be more significant. This is, for instance, the case of an estimation of a ship position sitting on land. Future work will include the study and mitigation of the systematic effects of the use of homographies, to improve the accuracy of the method. Furthermore, a future approach will make use of deep learning for the identification of the georeferenced pixel from the masks to minimise the presented georeferencing results. The use of deep learning will include the manual annotation of the pixel to be georeferenced from all the masks of the dataset as ground truth. The manual annotation of the pixel to be georeferenced can also be used as a baseline to analyse how good humans are at defining the pixel of a ship to be georeferenced against an automatic approach like the one we propose in our work. Further considerations will also include the automatic calculation of other ship parameters such as ship length, since the corresponding ground truth values are already available within the dataset. Future improvements of the dataset will also be shared with the public.

Our georeferencing method offers several improvements when compared with the state of the art in ship georeferencing from static oblique view images [29]. Firstly, our methodology can be replicated using any existing static camera at a maritime infrastructure without previous knowledge about the camera, such as location, elevation, field of view and tilt angle. Moreover, our quantitative analysis includes seven classes of ships and many ship sizes, from small boats to large container ships. We also analysed results in two independent ranges, within and over 400 m.

The ship segmentation and gereferencing method presented in this work is intended to be utilised as part of a complete pipeline for ship segmentation and georeferencing that can be used to present meaningful real-time information about ships to maritime situational awareness operators.

## 5. Conclusions

A novel dataset, ShipSG, for ship segmentation and georeferencing using a static oblique view of a port has been presented. This dataset contains images with mask annotations of ships present, and their corresponding class, position and length.

Four instance segmentation methods to recognise ships were explored using the dataset. DetectoRS shows the best overall mAP, though Centermask-Lite_V39_ is found to be the most precise of the real-time capable methods studied and is therefore most suited for our application. After training with ShipSG, the generalisation on a mini-dataset made of existing maritime datasets is shown.

A quantitative analysis of our homography based method for ship georeferencing from their segmented masks has also been presented. As expected, the accuracy of the method is best when the ship is closest to the camera. However, results prove that this is a reliable approach for all ship lengths independently from the range, since the georeferenced pixel is shown to fall within the bounds of the ship when considering both shorter and longer camera ranges.

Future studies will focus on the improvement of Centermask-Lite_V39_ to detect larger objects, the study of systematic effects of homographies for georeferencing, the use of deep learning to improve the identification of the pixel from the mask to be georeferenced and the integration of both tasks on a single pipeline that can be used by a maritime situational awareness system.

## Figures and Tables

**Table 1:** Number of masks annotated per class for each area scale.

Class	Small	Medium	Large	Total	%
Cargo	98	902	300	1300	11.18
Law Enforcement	101	3536	111	3748	32.24
Passenger/Pleasure	48	485	93	626	5.38
Special 1	64	427	511	1002	8.62
Special 2	265	2312	53	2630	22.62
Tanker	277	753	382	1412	12.15
Tug	249	470	188	907	7.80
**All classes**	**1102**	**8885**	**1638**	**11625**	**100**

**Table 2:** Configurations during training for each method evaluated.

Method	Input Size (Pixel)	Backbone	Batch Size	Iterations	Number of Epochs
Mask R-CNN [15]	1333 × 800	ResNeXt-101	2	15,400	11
DetectoRS [16]	1333 × 800	ResNet-50	2	15,400	11
YOLACT_550_ [17]	550 × 550	ResNet-50	8	6480	18
YOLACT_700_ [17]	700 × 700	ResNet-101	8	5760	16
Centermask-Lite_V19_ [18]	800 × 600	Vovnet-19	8	5949	17
Centermask-Lite_V39_ [18]	800 × 600	Vovnet-39	8	5949	17

**Table 3:** Resulting instance segmentation APs and inference speed per method evaluated. The two first rows are robust methods and the rest are the real-time methods.

Method	AP_ca_	mAP	mAP_50_	mAP_75_	mAP_S_	mAP_M_	mAP_L_	FPS
Mask R-CNN [15]	0.772	0.733	0.961	0.914	0.503	0.752	0.772	8.50
DetectoRS [16]	0.780	0.747	0.982	0.924	0.556	0.757	0.792	6.62
YOLACT_550_ [17]	0.571	0.527	0.886	0.609	0.086	0.515	0.709	36.28
YOLACT_700_ [17]	0.622	0.582	0.911	0.700	0.140	0.582	0.751	27.75
Centermask-Lite_V19_ [18]	0.732	0.635	0.840	0.780	0.455	0.640	0.657	40.98
Centermask-Lite_V39_ [18]	0.740	0.644	0.839	0.787	0.461	0.648	0.661	35.25

**Table 4:** Generalisation mask AP results per model, the first two rows are robust methods and the rest are real-time capable methods. All the classes have been contemplated as a single class (class-agnostic).

Method	AP	AP_50_
Mask R-CNN [15]	0.441	0.749
DetectoRS [16]	0.486	0.830
YOLACT_550_ [17]	0.340	0.615
YOLACT_700_ [17]	0.336	0.613
Centermask-Lite_V19_ [18]	0.348	0.636
Centermask-Lite_V39_ [18]	0.387	0.710

**Table 5:** Quantitative ship georeferencing results. Δφ and Δλ stand for absolute latitude and longitude error, respectively. GDE stands for georeferencing distance error. Std stands for standard deviation. DRSME stands for distance root-mean-square error.

Location	Mean Δφ [m]	Mean Δλ [m]	Mean GDE [m]	Std GDE [m]	DRSME [m]
Port Basin (range < 400 m)	16	12	22	10	27
River (range > 400 m)	42	27	53	24	61

## Data Availability

The data presented and analysed in this work can be found here: https://dlr.de/mi/shipsg, accessed on 16 February 2022.

## Abbreviations

The following abbreviations are used in this manuscript:

VTS	Vessel Traffic Services
AIS	Automatic Identification System
SMD	Singapore Maritime Dataset
DLR	German Aerospace Center
AP	Average Precision
mAP	mean Average Precision
FPS	Frames Per Second
UTM	Universal Transverse Mercator
GDE	Georeferencing Distance Error
DRMSE	Distance Root-Mean-Square Error

## Appendix A. Class Average Precision per Class and Instance Segmentation Method

This appendix contains the resulting instance segmentation APs per class and method explored in Section 2.2.

**Table A1:** Resulting instance segmentation APs per class of the ShipSG dataset using Mask-RCNN, as explained in Section 3.1.

Class	AP	AP_50_	AP_75_	AP_S_	AP_M_	AP_L_
Cargo	0.707	0.987	0.938	0.541	0.715	0.762
Law Enforcement	0.842	0.99	0.978	0.558	0.868	0.73
Passenger/Pleasure	0.705	0.951	0.919	0.542	0.737	0.72
Special 1	0.754	0.947	0.923	0.357	0.722	0.83
Special 2	0.773	0.958	0.927	0.51	0.808	0.81
Tanker	0.677	0.973	0.86	0.52	0.685	0.757
Tug	0.671	0.921	0.851	0.491	0.728	0.797

**Table A2:** Resulting instance segmentation APs per class of the ShipSG dataset using DetectoRS, as explained in Section 3.1.

Class	AP	AP_50_	AP_75_	AP_S_	AP_M_	AP_L_
Cargo	0.718	0.995	0.933	0.59	0.711	0.788
Law Enforcement	0.848	0.99	0.979	0.563	0.869	0.778
Passenger/Pleasure	0.733	0.983	0.952	0.62	0.756	0.761
Special 1	0.786	0.975	0.944	0.466	0.739	0.847
Special 2	0.772	0.985	0.935	0.584	0.799	0.814
Tanker	0.687	0.994	0.845	0.534	0.695	0.767
Tug	0.685	0.949	0.882	0.539	0.728	0.791

**Table A3:** Resulting instance segmentation APs per class of the ShipSG dataset using YOLACT_550_, as explained in Section 3.1.

Class	AP	AP_50_	AP_75_	AP_S_	AP_M_	AP_L_
Cargo	0.469	0.889	0.424	0.08	0.438	0.722
Law Enforcement	0.685	0.978	0.91	0.072	0.703	0.717
Passenger/Pleasure	0.512	0.887	0.589	0.11	0.546	0.633
Special 1	0.617	0.906	0.771	0.039	0.446	0.745
Special 2	0.51	0.933	0.552	0.155	0.545	0.716
Tanker	0.442	0.82	0.504	0.065	0.454	0.668
Tug	0.454	0.792	0.511	0.079	0.471	0.766

**Table A4:** Resulting instance segmentation APs per class of the ShipSG dataset using YOLACT_700_, as explained in Section 3.1.

Class	AP	AP_50_	AP_75_	AP_S_	AP_M_	AP_L_
Cargo	0.529	0.911	0.54	0.13	0.506	0.728
Law Enforcement	0.727	0.978	0.923	0.123	0.745	0.76
Passenger/Pleasure	0.577	0.896	0.768	0.197	0.595	0.728
Special 1	0.669	0.937	0.809	0.076	0.529	0.782
Special 2	0.57	0.947	0.739	0.203	0.609	0.775
Tanker	0.508	0.901	0.559	0.137	0.536	0.704
Tug	0.494	0.808	0.558	0.112	0.551	0.78

**Table A5:** Resulting instance segmentation APs per class of the ShipSG dataset using Centermask-Lite_V19_, as explained in Section 3.1.

Class	AP	AP_50_	AP_75_	AP_S_	AP_M_	AP_L_
Cargo	0.597	0.848	0.767	0.497	0.585	0.644
Law Enforcement	0.739	0.853	0.854	0.520	0.763	0.626
Passenger/Pleasure	0.608	0.844	0.778	0.484	0.627	0.625
Special 1	0.661	0.843	0.798	0.313	0.571	0.719
Special 2	0.665	0.838	0.798	0.429	0.686	0.674
Tanker	0.576	0.832	0.730	0.438	0.589	0.618
Tug	0.601	0.824	0.736	0.504	0.657	0.695

**Table A6:** Resulting instance segmentation APs per class of the ShipSG dataset using Centermask-Lite_V39_, as explained in Section 3.1.

Class	AP	AP_50_	AP_75_	AP_S_	AP_M_	AP_L_
Cargo	0.598	0.823	0.784	0.477	0.594	0.638
Law Enforcement	0.731	0.835	0.843	0.498	0.756	0.612
Passenger/Pleasure	0.607	0.825	0.779	0.517	0.629	0.622
Special 1	0.652	0.818	0.785	0.269	0.555	0.733
Special 2	0.657	0.826	0.786	0.436	0.676	0.684
Tanker	0.565	0.815	0.732	0.442	0.578	0.616
Tug	0.699	0.93	0.799	0.59	0.752	0.721
